# A Triple-Masked, Randomized Controlled Trial Comparing Ultrasound-Guided Brachial Plexus and Distal Peripheral Nerve Block Anesthesia for Outpatient Hand Surgery

**DOI:** 10.1155/2014/324083

**Published:** 2014-04-15

**Authors:** Nicholas C. K. Lam, Matthew Charles, Deana Mercer, Codruta Soneru, Jennifer Dillow, Francisco Jaime, Timothy R. Petersen, Edward R. Mariano

**Affiliations:** ^1^Department of Anesthesiology & Critical Care Medicine, University of New Mexico, Albuquerque, NM 87131, USA; ^2^Department of Orthopaedics & Rehabilitation, University of New Mexico, Albuquerque, NM 87131, USA; ^3^Department of Anthropology, University of New Mexico, Albuquerque, NM 87131, USA; ^4^Department of Anesthesiology, Perioperative and Pain Medicine, Stanford University School of Medicine, Stanford, CA, USA; ^5^Anesthesiology and Perioperative Care Service, Veterans Affairs Palo Alto Health Care System, 3801 Miranda Avenue (112A), Palo Alto, CA 94304, USA

## Abstract

*Background*. For hand surgery, brachial plexus blocks provide effective anesthesia but produce undesirable numbness. We hypothesized that distal peripheral nerve blocks will better preserve motor function while providing effective anesthesia. *Methods*. Adult subjects who were scheduled for elective ambulatory hand surgery under regional anesthesia and sedation were recruited and randomly assigned to receive ultrasound-guided supraclavicular brachial plexus block or distal block of the ulnar and median nerves. Each subject received 15 mL of 1.5% mepivacaine at the assigned location with 15 mL of normal saline injected in the alternate block location. The primary outcome (change in baseline grip strength measured by a hydraulic dynamometer) was tested before the block and prior to discharge. Subject satisfaction data were collected the day after surgery. *Results*. Fourteen subjects were enrolled. Median (interquartile range [IQR]) strength loss in the distal group was 21.4% (14.3, 47.8%), while all subjects in the supraclavicular group lost 100% of their preoperative strength, *P* = 0.001. Subjects in the distal group reported greater satisfaction with their block procedures on the day after surgery, *P* = 0.012. *Conclusion*. Distal nerve blocks better preserve motor function without negatively affecting quality of anesthesia, leading to increased patient satisfaction, when compared to brachial plexus block.

## 1. Introduction

Ultrasound-guided regional anesthesia is commonly performed for patients undergoing hand surgery [1]. However, the inability to use the affected limb due to motor block has been shown to reduce patient satisfaction [2, 3]. To address this issue, alternative regional anesthesia techniques have been suggested [4, 5]; for wrist and hand surgery, one approach involves short-acting brachial plexus block combined with long-acting distal peripheral nerve blocks [6, 7]. Unfortunately, this approach does not avoid the necessary period of immobility caused by a proximal brachial plexus block and may not improve patient satisfaction [8].

For outpatient hand surgery, distal peripheral nerve blocks alone should preserve motor function and may provide effective anesthesia [9, 10], thereby leading to greater patient satisfaction with the regional anesthetic technique and the surgical experience. However, the degree of motor sparing with this technique has not been established yet. We designed this randomized, triple-masked, clinical trial to test the primary hypothesis that ultrasound-guided distal peripheral nerve blocks in the forearm will preserve motor function to a greater extent than ultrasound-guided supraclavicular blocks while holding total local anesthetic dose constant.

## 2. Materials and Methods

The Human Research Review Committee at the University of New Mexico Health Sciences Center (Albuquerque, NM) approved this study, and the protocol was registered prospectively with ClinicalTrials.gov (NCT01927289). All subjects provided written informed consent.

Eligible subjects were 18 years or older, ASA I to III, and scheduled for elective ambulatory hand surgery with an expected surgical time of less than 15 minutes under regional anesthesia and intravenous sedation. Exclusion criteria were surgery outside the median and ulnar nerve distributions, bilateral surgery, body mass index of more than 45 kg/m^2^, neck or clavicle deformities, infection at the planned injection site, existing neurologic disease, chronic pain diagnosis, opioid use greater than two weeks in the last six months, insulin dependent diabetes mellitus or diabetes with end-organ damage, allergy to local anesthetics, coagulopathy, previous surgery on the operative limb, and inability to comprehend study-related procedures.

### 2.1. Randomization

Randomization for group allocation was performed using a computer-generated random sequence (http://www.random.org/) to produce a series of sealed envelopes containing allocation instructions. One envelope was selected for each subject after consent was obtained and the unopened allocation envelope was immediately delivered to the anesthesiologist responsible for preparing study drugs. This anesthesiologist was not involved in any subsequent study procedures. Each subject was randomly assigned to one of two groups: distal (distal peripheral nerve blocks) or proximal (supraclavicular block). Regardless of group assignment, all subjects received the same volume and concentration of local anesthetic to keep dose constant, 15 mL of 1.5% mepivacaine at the assigned injection site with 15 mL of normal saline injected in the alternate location. The order of procedures was the same for all subjects: distal peripheral nerve injections of masked study solution first followed by proximal supraclavicular injection of masked study solution.

### 2.2. Masking

After informed consent was obtained, a 20 mL syringe containing 15 mL of 1.5% mepivacaine and a 20 mL syringe containing 15 mL of normal saline were filled and labeled as to block site according to the allocation. This preparation was performed by an anesthesiology provider not involved in block administration, care of the subject, or assessment. The subject, regional anesthesiologist performing the block, assessor, all health care providers, and the data analyst were masked to the allocation.

### 2.3. Patient and Procedural Preparation

All subjects were given celecoxib 400 mg orally before surgery unless contraindicated. All block procedures were performed preoperatively in a holding room by a single regional anesthesiologist trained in both proximal brachial plexus and distal peripheral nerve blocks under ultrasound guidance. Subjects' motor function was assessed by testing their hand grip strength using a hydraulic hand dynamometer (JAMAR^©^, Lafayette Instrument Company, Lafayette, IL). Three baseline measurements were recorded on both the ipsilateral and contralateral sides. Before nerve blockade, all subjects received intravenous access, supplemental oxygen, and standard monitoring which included noninvasive blood pressure, electrocardiogram, and pulse oximetry. Sedation and anxiolysis were achieved with intravenously administered midazolam in 1 to 2 mg increments and fentanyl in 25 mcg increments as needed for subject comfort.

### 2.4. Distal Peripheral Nerve Blocks

#### 2.4.1. Ulnar Nerve

The subject was placed supine with the ipsilateral arm abducted and externally rotated with the palm facing up. The palmar and medial side of the ipsilateral forearm was disinfected using chlorhexidine gluconate (ChloraPrep; CareFusion, San Diego, CA). A 28 mm high-frequency ultrasound probe (L28, S-Nerve, Fujifilm SonoSite Inc., Bothell, WA) covered with a sterile dressing was placed at the mid-forearm level to identify the flexor digitorum superficialis muscles and flexor digitorum profundus muscles where the median nerve is located [11]. A long axis slide was used to trace the median nerve distally to 5 cm from the proximal wrist crease [10]. A short axis slide was then performed medially to locate the ulnar artery and the ulnar nerve adjacent to it [12]. Upon location of these structures, 1.5 mL of 0.5% lidocaine with 5 mcg/mL epinephrine was injected subcutaneously at the medial border of the ultrasound probe. This was the point of needle insertion for both ulnar and median nerve block [10]. A 22 gauge 80 mm insulated echogenic needle (SonoPlex Stim Cannula; Pajunk Medizintechnologie, Germany) was inserted through the skin at the medial border of the probe and advanced in plane from medial to lateral toward the ulnar nerve. Five mL of masked study solution was injected around the ulnar nerve, and circumferential spread was confirmed by ultrasound.

#### 2.4.2. Median Nerve

**Figure 1:**
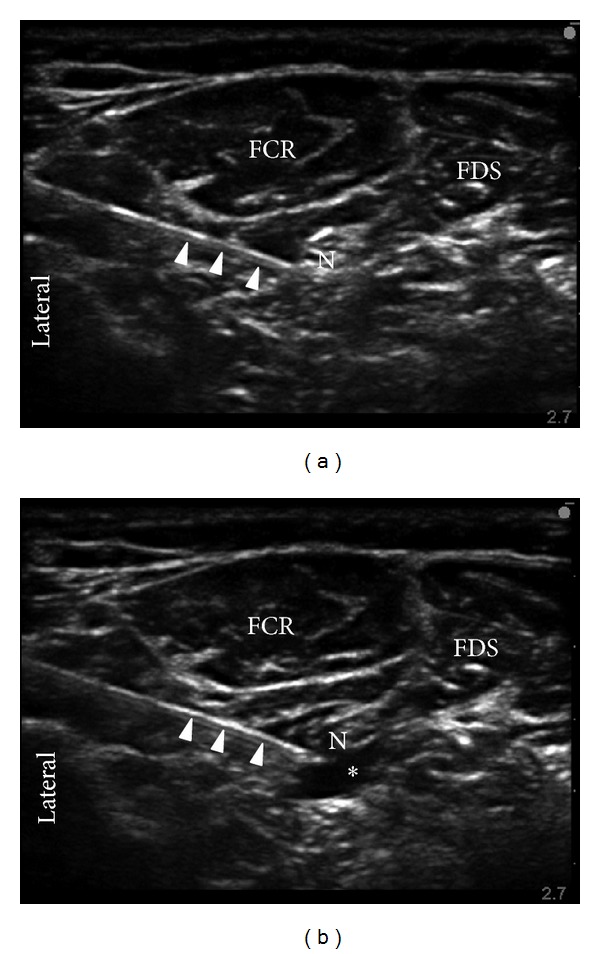
Short-axis sonograms of ultrasound-guided median nerve block at two time points: preinjection (a) and postinjection (b); arrows identify the needle; N = nerve; FCR = flexor carpi radialis muscle; FDS = flexor digitorum superficialis muscle; ∗ = injectate solution.

Following ulnar nerve injection, a short axis slide laterally allowed for the visualization of the median nerve at the same level as the injection point for the ulnar nerve. The same needle was then redirected further laterally towards the median nerve using an in-plane approach. Five mL of masked study solution was injected around the median nerve (Figure 1). Redirection of the needle at the discretion of the regional anesthesiologist was allowed to achieve circumferential spread [10]. At the end of the ulnar and median nerve block procedures, 5 mL of masked study solution was used to raise a wheal on the volar side of the wrist at the level of the palmar crease.

### 2.5. Supraclavicular Block

Supraclavicular brachial plexus block was performed with the subject supine with the head of the bed slightly elevated. The anticipated block site in the ipsilateral supraclavicular fossa was prepared using the same antiseptic precautions as the distal group, and the same ultrasound equipment was used. With the ultrasound transducer placed in the supraclavicular fossa in a coronal-oblique plane posterior to the clavicle, the brachial plexus was identified in proximity to the subclavian artery above the first rib [13]. The first rib and pleura were identified in all subjects. After subcutaneous infiltration of 3 mL of a premixed commercially available 0.5% lidocaine with 5 mcg/mL epinephrine at the planned insertion site lateral to the ultrasound transducer, the same type of needle used in the distal group was inserted and advanced in plane toward the brachial plexus [13]. The needle tip was positioned under US guidance lateral to the subclavian artery and cephalad to the first rib (i.e., “corner pocket”) adjacent to the hypoechoic neural structures of the brachial plexus [13, 14]. After negative aspiration, 15 mL of the masked study solution was injected incrementally to ensure circumferential spread; 5 mL was injected in the corner pocket with the remaining 10 mL distributed evenly within the neural compartment to produce circumferential spread around the plexus.

The procedural time (seconds) for each block was recorded. This duration was defined for the individual block procedure as the interval from the ultrasound transducer's first contact with the subject to the time the block needle exited the skin. Total procedural time was defined as the interval from first ultrasound transducer contact for the first distal block to completion of the supraclavicular block with removal of the last block needle.

### 2.6. Block Assessment

After completion of all block procedures, sensory and motor blockade were evaluated every five minutes for 30 minutes. Data collection was performed by an independent observer who was masked to each subject's group allocation. The extent of sensory blockade was tested in the median nerve and ulnar nerve distribution of the hand using ice on the palmar surface of the index finger and the little finger, respectively: 0 = no perception, 1 = decreased sensation, or 2 = normal sensation. Successful blockade was defined as complete sensory blockade (i.e., sensory block score = 0) in both peripheral nerve distributions within 30 minutes of completing the supraclavicular block.

### 2.7. Perioperative Care

If complete sensory blockade was not achieved within 30 minutes, the affected subject was excluded from the study and categorized as a block failure. The subject was offered a supplementary nerve block, if there was sufficient time prior to surgery, or general anesthesia to achieve surgical anesthesia. A single upper arm pneumatic tourniquet was used for all subjects. Intravenous sedation using midazolam and/or propofol were provided intraoperatively as per routine clinical practice and titrated to the comfort of the subject, while maintaining verbal contact. Short-acting opioids were given intraoperatively only if the subject complained of pain. No other anesthetics or analgesics, such as dexmedetomidine, ketamine, ketorolac, or intravenous acetaminophen, were given intraoperatively. Any subject who required moderate or deep sedation, general anesthesia, or supplementary blocks was noted. After surgery, 5 mL of 0.25% bupivacaine incisional infiltration was performed by the surgeon in all subjects as per routine clinical practice. Postoperatively, all subjects were prescribed intravenous and oral opioids as needed for breakthrough pain in the postanesthesia care unit (PACU). In the PACU, three hand grip strength measurements were recorded for all subjects using the same dynamometer on both the ipsilateral and contralateral sides. Subjects were discharged from PACU to home with prescriptions for oral ibuprofen 400 mg and two tablets of hydrocodone 5 mg with acetaminophen 325 mg to be taken every four hours as needed for pain. Subjects were advised to consume both analgesics when they first felt the hand numbness resolving.

### 2.8. Outcomes

The primary outcome of this study was postoperative change in hand grip strength in the operative limb as a percent reduction of preoperative baseline strength. Secondary outcomes on the day of surgery included procedural time, block success/failure, onset time, block duration, sedative and analgesic requirements, and surgery duration. Each surgeon was interviewed by an assessor blinded to the allocation of the subject at the end of surgery to assess his/her satisfaction using a seven-point Likert scale: “On a scale of 1 to 7 with 1 being* not at all*, 4 being* neutral*, and 7 being* completely satisfied*, how satisfied were you with the surgical conditions provided for this subject?”

On postoperative day one, subjects were interviewed by an assessor blinded to group allocation regarding their satisfaction with the block via telephone. Subject satisfaction was assessed with a standardized question scored on a seven-point Likert scale: “Thinking about your nerve blocks, how satisfied were you with them: where 1 is* not at all*, 4 is* neutral*, and 7 is* completely satisfied*?” The interview also included inquiries on the time that the subjects first felt the return of sensation and the time they first felt full recovery of strength. Subjects were asked about the occurrence of any adverse events or potential block-related complications, including paresthesias, motor deficits, persistent pain, and bruising. If complications occurred, they were followed up as per routine clinical practice and noted for the study.

### 2.9. Sample Size Estimate

Based on a pilot study performed at the authors' institution (unpublished data), we assumed that the distal block group will experience a 10% decrease in hand grip strength from baseline compared to a 90% decrease in the supraclavicular block group. Assuming a similar effect size, 80% power, and *α* = 0.05 using a two-sample test of proportions, we estimated that seven subjects per group would be required.

### 2.10. Statistical Analysis

Normality of data distribution was determined using the Shapiro-Wilk test. Continuous data with normal distribution were presented as mean (standard deviation [SD]) and analyzed with Student's *t*-test; continuous data with nonnormal distribution were presented as median (interquartile range [IQR]) and analyzed using the Mann-Whitney *U* test. Categorical data were analyzed with the Chi square test or Fisher's exact test (*n* < 5 in any field). Correlation between change in hand grip strength and patient satisfaction was assessed with two nonparametric measures of association: Spearman's rho and Kendall's tau. For the primary outcome, *P* < 0.05 was considered statistically significant. The results from secondary outcome analyses should be interpreted as suggestive and not conclusive.

## 3. Results

**Figure 2:**
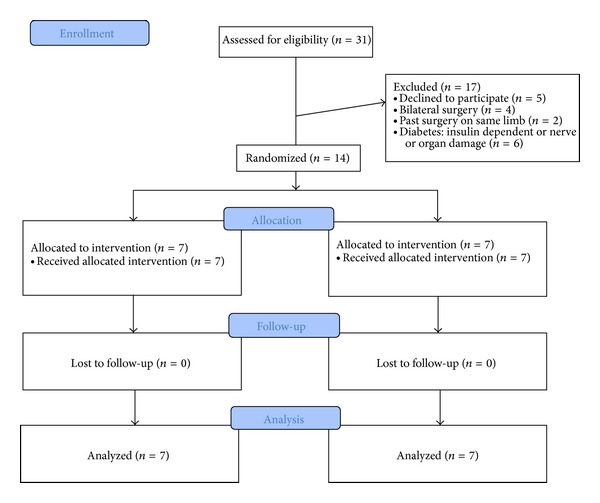
CONSORT study flow diagram.

Thirty-one patients were assessed for eligibility and offered enrollment in this study. Five eligible patients refused to participate, and 12 were excluded based on study criteria (e.g., bilateral surgery, previous surgery on the operative limb, and diabetes with nerve or end organ damage), leaving 14 subjects who were enrolled and successfully completed study procedures (Figure 2).

Demographic and surgical data are presented in Table 1; no statistically significant differences in these variables were noted between study groups. All subjects received preoperative celecoxib except for one subject in the proximal group with sulfur allergy. There were no failed blocks in either group, and no subjects required breakthrough analgesia or experienced postoperative nausea or vomiting in the PACU.

### 3.1. Primary Outcome

Median (IQR) strength loss in the surgical limb for the distal group was 21.4% (14.3%, 47.8%), while all subjects in the proximal group lost 100% (100%, 100%) of their preoperative strength, *P* = 0.001.

### 3.2. Secondary Outcomes

Block procedures in the proximal group required less time [mean (SD)] than in the distal group: 226.8 (38.5) versus 408.8 (82.3) seconds, respectively (*P* < 0.0001; 95% CI for difference 131.0–232.9 seconds). Subjects in the distal group reported higher satisfaction scores [median (interquartile range)] with their block procedures than those in the proximal group on the day after surgery: 7 (7, 7) versus 6 (6, 6), respectively (*P* = 0.012). Subject satisfaction correlated inversely with operative limb strength loss based on Spearman's rho −0.62 (*P* = 0.016) and Kendall's tau is −0.55 (*P* = 0.025). Subject-reported mean (SD) time to return of sensation in the distal group was 261 (91.6) minutes, while that in the proximal group was 358 (53.5) minutes (*P* = 0.03, 95% CI for the difference: 40.1–96.4 minutes). There were no differences in other secondary outcomes (Table 2).

One subject exhibited symptoms of persistent paresthesia in the proximal group, but this had resolved by the second postoperative day. There were no protocol violations or other adverse events.

## 4. Discussion

Both ultrasound-guided distal peripheral nerve blocks and proximal brachial plexus blocks can be used as the primary anesthetic for outpatient hand surgery, but distal peripheral nerve blocks are superior at preserving motor function of the operative limb and may lead to modest improvements in patient satisfaction.

Unlike the present study, Fredrickson and colleagues did not find a difference in subject satisfaction when the combination of short-acting brachial plexus block and distal nerve blocks was compared to long-acting brachial plexus block; however, the median satisfaction score on a 0–10 scale (with 10 = “very satisfied overall”) was 10 in each group [8]. Since all subjects in the Fredrickson study experienced complete upper extremity motor block, albeit of different duration, and would have expected this type of block postoperatively, we speculate that these pain scores reflect managed expectations. Perhaps, if presented with the alternative of avoiding motor block, these satisfaction results may have been different. We designed the present study based on this assumption, given the published feasibility of distal peripheral nerve blocks as an anesthetic technique for minor hand surgery [15–17], and subjects in our distal group did experience less motor block and subsequently rated their satisfaction higher than the proximal group. The present study is the first to rigorously compare the anesthetic quality of the distal peripheral nerve block technique to an established standard (brachial plexus blockade).

### 4.1. Importance of Preserving Motor Function

The use of distal peripheral nerve blocks as a primary anesthetic technique for trigger finger or carpal tunnel release, with preservation of motor function, may allow patients to move the affected digit(s) or hand when instructed to do so during surgery. While local anesthetic infiltration alone [18] and intravenous regional anesthesia (IVRA or Bier block) [19] are also acceptable techniques for minor hand surgery, surgeons at our institution prefer distal peripheral nerve blocks to avoid local anesthetic-induced distortion of the surgical field. Distal peripheral nerve blocks have been associated with shorter surgical time when compared to IVRA for carpal tunnel release [9]. One potential patient safety advantage of distal peripheral nerve blocks is avoiding the upper limb immobility and lack of protective reflexes resulting from brachial plexus blockade especially for outpatients. Studies on ambulatory brachial plexus catheter patients with lower local anesthetic concentrations have not been able to eliminate self-reported numbness [20, 21]. Avoiding motor block through the use of distal peripheral nerve blocks alone eliminates the need for a sling (and its associated cost) and may allow patients to better protect the operative limb from inadvertent injury [22].

### 4.2. System Considerations

The procedural time for supraclavicular block is shorter than that for distal peripheral nerve blocks by three minutes on average, consistent with previous-published procedural times [9]. However, the total anesthesia preparation time combining block performance time and time for anesthetic onset is similar between groups; therefore, this minor procedural time difference does not appear to be clinically relevant.

### 4.3. Local Anesthetic Dosing

The total local anesthetic dosage in the present study was held constant between groups so any difference in postoperative hand grip strength could not be attributed to a difference in dose. A previous study has shown that the minimum effective volume (MEV) of 1.5%* lidocaine* for ultrasound-guided supraclavicular block when injecting in the corner pocket and superior compartment is 30 mL [23]. Compared to our study, the study by Tran and colleagues enrolled a sample of patients undergoing a more diverse set of surgical procedures (hand, wrist, forearm, and elbow) versus strictly minor hand surgery and used lidocaine instead of mepivacaine [23]. A more recent study of ultrasound-guided supraclavicular blocks using 1.5% mepivacaine, although the technique slightly differs from the one used in the present study, has shown that the MEV90 (estimated for 90% of patients) is 15 mL—the same volume used in the present study [24]. No subjects in the proximal group had failed blocks or required analgesics in the recovery room; therefore, we conclude that the observed difference in patient satisfaction is not likely related to a difference in anesthetic efficacy.

### 4.4. Study Limitations

Only surgeries of brief duration (<15 minutes expected surgical time) were included in our study in order to minimize tourniquet pain which would not be covered by the distal forearm blocks. Regarding the primary outcome of hand grip strength, we recognize that performance is dependent on subject effort; if a subject is tired or distracted, he or she may not perform as well on a trial even though the same innate muscle strength is present. We attempted to minimize this variability by taking three measurements at each assessment and by blinding the assessor and providers. The results of the present study may only apply to practices employing similar regional anesthetic and surgical techniques and equipment; in particular, this study did not involve the use of nerve stimulation, so we do not know how this nerve localization modality may have influenced outcomes. Finally, the use of a second sham block in all subjects for the purposes of masking is not a part of usual clinical practice; eliminating the sham block can be expected to reduce overall procedural time.

## 5. Conclusions

In summary, this study suggests that ultrasound-guided distal peripheral nerve blocks can be an effective alternative to brachial plexus blockade as the primary anesthetic for outpatient hand surgery and offers the potential advantage of preserved motor function.

## Figures and Tables

**Table 1:** Demographic and surgical observations.

	Distal group (*n* = 7)	Proximal group (*n* = 7)	*P* value
Age, years	54.0 (5.1)	53.7 (5.6)	0.92
Sex, *n* (m/f)	3/4	3/4	0.99
Height, cm	163.0 (9.1)	164.7 (11.8)	0.77
Weight, kg	86.5 (16.2)	87.6 (17.1)	0.90
Surgery duration, min	12.1 (2.6)	15.7 (4.5)	0.09
Tourniquet duration, min	8.0 (2.2)	10.0 (3.4)	0.21
Surgeon's satisfaction, 1–7	7 (7, 7)	7 (7, 7)	0.39

Continuous data are presented as mean (SD) when normally distributed or median (interquartile range) when not normally distributed; count data are presented as number of subjects as appropriate.

**Table 2:** Secondary outcomes observations.

	Distal group (*n* = 7)	Proximal group (*n* = 7)	*P* value
Anesthetic onset time, min	5 (5, 15)	10 (10, 20)	0.12
Bruising, *n*	3	2	0.99
Paresthesia, *n*	0	1	0.99
Total anesthesia preparation time (both blocks + onset time), min	16.1 (14.8, 26.0)	21.8 (18.5, 28.8)	0.20
Assigned block procedural time + onset time, min	12.1 (11.0, 22.2)	14.8 (13.5, 22.8)	0.31

Data are presented as median (interquartile range) or number of subjects, as appropriate.
